# Finite Element Analysis of Interaction of Laser Beam with Material in Laser Metal Powder Bed Fusion Process

**DOI:** 10.3390/ma11050765

**Published:** 2018-05-10

**Authors:** Guang Fu, David Z. Zhang, Allen N. He, Zhongfa Mao, Kaifei Zhang

**Affiliations:** 1 State Key Laboratory of Mechanical Transmission, Chongqing University, Chongqing 400044, China; guangfu@cqu.edu.cn (G.F.); zhongfamao@cqu.edu.cn (Z.M.); kaifeizhang@cqu.edu.cn (K.Z.); 2 College of Engineering, Mathematics and Physical Sciences, University of Exeter, North Park Road, Exeter EX4 4QF, UK; n.he@exeter.ac.uk

**Keywords:** additive manufacturing, laser powder bed fusion, metal powder, finite element analysis, absorption, temperature distribution

## Abstract

A deep understanding of the laser-material interaction mechanism, characterized by laser absorption, is very important in simulating the laser metal powder bed fusion (PBF) process. This is because the laser absorption of material affects the temperature distribution, which influences the thermal stress development and the final quality of parts. In this paper, a three-dimensional finite element analysis model of heat transfer taking into account the effect of material state and phase changes on laser absorption is presented to gain insight into the absorption mechanism, and the evolution of instantaneous absorptance in the laser metal PBF process. The results showed that the instantaneous absorptance was significantly affected by the time of laser radiation, as well as process parameters, such as hatch space, scanning velocity, and laser power, which were consistent with the experiment-based findings. The applicability of this model to temperature simulation was demonstrated by a comparative study, wherein the peak temperature in fusion process was simulated in two scenarios, with and without considering the effect of material state and phase changes on laser absorption, and the simulated results in the two scenarios were then compared with experimental data respectively.

## 1. Introduction

The powder bed fusion (PBF) process is one of the earliest commercialized additive manufacturing (AM) processes, and is also the most popular one, attracting more and more attention from industrial practitioners [1]. During the PBF process, a thin loose powder layer is evenly spread over a solid substrate by a recoater blade, and then a laser or electron beam energy source is used to selectively fuse and consolidate the powder layer to achieve a desired layer geometry. When one layer is completed, the building platform is lowered by a defined layer thickness, a fresh layer of powder is spread again, and the process is repeated layer-by-layer until a 3D object is produced. To date, the PBF process has been successfully applied to fabricate parts made from a wide range of materials including polymers, metals, ceramics, and composites [1]. The major binding mechanisms used in PBF include solid-state sintering, chemically-induced binding, liquid-phase sintering, and full melting [2], out of which the full melting mechanism is commonly and broadly used in commercialized PBF-based metallic AM systems to fabricate high-density parts, without the need for post-process densification [3].

Laser metal PBF is a complex manufacturing process involving a series of physical phenomena [4,5]. In this process, a laser beam initially interacts with metal powder, which causes the powder to undergo state and phase changes from powder to liquid, and then to solid. Heat is transferred from the zone of laser-material interaction into the surrounding powder and the underlying substrate through conduction and radiation, and to the ambient environment through radiation and convection. Temperature gradients are developed between the center and edge of the pool of metal liquid melted by laser, which give rise to a variation of surface tension over the pool. This variation induces a Marangoni flow from a region of low surface tension to a region of high surface tension. During the PBF process, the variation of surface tension may result in a wetting or balling effect of the metal liquid pool, and the evaporation of metal liquid due to high energy density input may also suppress temperature. These phenomena have a profound influence on the quality properties of the finished parts. However, experimental approaches to understanding the phenomena (i.e., laser-material interaction and temperature gradients) have limitations due to the high cost of measurement and the highly transient nature of the process. In this sense, numerical methods become the most feasible way to investigate how the aforementioned phenomena affect the properties of the produced parts, such as microstructure and thermal cracks.

**Figure 1:**
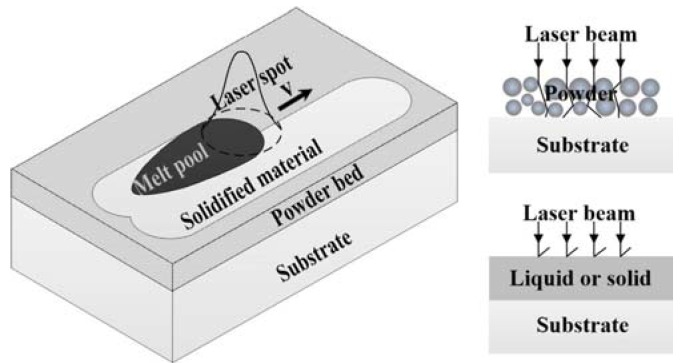
Schematic of absorption mechanism during laser metal powder bed fusion (PBF) process.

The finite element (FE) method, a numerical method, has been widely used in recent years. FE models [6,7,8,9,10,11,12,13,14,15,16,17,18,19,20,21,22,23,24,25] have been developed for investigating different laser metal PBF processes, including selective laser sintering (SLS) [7,8,9,10], selective laser melting (SLM) [12,13,14,16,17,18,19,20,21,22,23,24], laser micro-sintering (LMS) [11], and multi-materials laser densification (MMLD) [15,25]. Some models are devoted to the analysis of the temperature distribution from the surface of the powder bed to the substrate [21], whilst others are developed to predict the evolution of transient temperature [8,17,18,19,20], residual stresses [15,16,17,18,26,27], and distortions [15] of the fabricated parts. In the literature, there are FE-based experiential studies which investigate the effects of process parameters and material properties in the PBF process on the quality of finished parts, such as laser power [7,9,10,11,12,13,14], scanning velocity [9,10,11,12,13,14,15], line energy [22,24], beam size [7,9,10,11], hatch space [7,10,12,24], layer thickness [15], track length [24], scanning patterns [6,23], volume shrinkage due to phase change from material in the powder state to the liquid state and to solid state [24,25], interval time between neighboring tracks [24], preheating temperature [9,10,15], and powder porosity [15]. However, many studies ignore the effect of material state and phase changes on laser absorption when modelling the interaction between laser beam and materials, and consider the absorptance as constant. However, the real fact is that the laser energy is absorbed by material in multiple states [4], namely powder, liquid, and solid as shown in Figure 1, and the absorptance varies according to the time of laser radiation and process parameters, such as scanning velocity, hatch space, and laser power in the PBF process [28,29,30,31]. This can be further clarified as a fact that the surface area of the liquid, solidified, and powder material covered by the laser spot change with the time of laser radiation and the process parameters mentioned above. Considering each state of material has a different absorption capacity due to a different absorption mechanism [32], a deep understanding of laser-material interaction mechanisms, characterized by laser absorption of material in multiple states, becomes a key issue for understanding the PBF process.

In this paper, a FE model of heat transfer considering the effect of material state and phase changes on laser absorption is presented to gain insight into the absorption mechanism and its evolution with the time of laser radiation, as well as hatch space, scanning velocity, and laser power. For the time being, we hereby neglect certain important phenomena, such as fluid convection in the melt pool and evaporation. Through comparison with the experimental data in peak temperature, our model, however, shows better results than the existing FE models without considering the effect of material state and phase changes on laser absorption.

## 2. Model Description

### 2.1. General Setup

**Figure 2:**
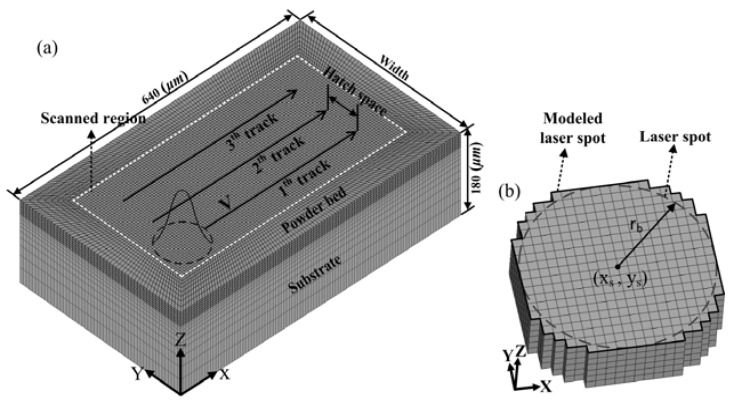
(**a**) 3D finite element model; (**b**) representation of the modeled laser spot. The modeled spot area is defined as Ω.

Three states and phases of material are considered, namely powder, liquid, and solid. In every computational time step, the applied heat source forms for a cell at laser spot location, containing surface heat source and volume heat source, and the specific laser absorption coefficient for this cell are based on an analysis of whether the cell remains as powder or becomes dense (i.e., liquid or solid). Figure 2a shows the geometry, scanning pattern, and mesh type of the model. A powder layer with a given thickness (30 μm) is evenly spread over the substrate. The substrate is preheated to a temperature at 35 °C (*T*_0_). An incoming heat source (i.e., a laser beam), *q_in_*, with a Gaussian distribution, moves at a specific scanning velocity in a parallel scanning strategy. The continuous movement of the heat source on the *X*-axis is divided into a number of steps, and the time duration of each step is defined by the size of the element and scanning velocity [18], as follows

(1) $$t_{step}=\frac{\Delta x}{V}$$

where *t_step_* is the time duration of a step, ∆*x* is the length of element, and *V* is the scanning velocity of the heat source. In order to enable the FE model to achieve an accurate result, every time step is further divided into several sub-time steps [20]. Once the solution of each time step is obtained, an analysis of whether an element remains as powder or becomes liquid is performed, which is based on the examination of the temperatures of all nodes of the element. If the temperatures of all nodes exceed the melting temperature of the material, the analysis result is “liquid”. Otherwise, it is “powder”. This process is repeated until a track is completed. Then, the heat source spends a given interval time (200 μs) to switch off and jump to a new track, which is usually a hatch space away from the previous scanned track.

The algorithm in the model consists of a sequential set of processing steps, including calculating the heat transfer equation, updating the material properties, determining the area distribution of each phase and state, as well as calculating the instantaneous absorptance at laser spot location in a new time step. These various steps will be discussed in detail in the following subsections.

### 2.2. Enthalpy Equation

The thermal transient spatial temperature distribution *T*(*x, y, z, t*) satisfies the following enthalpy equation given by Reference [4]

(2) $$\rho \frac{\partial H}{\partial t}=\nabla \cdot k\nabla T+q_{s}+q_{v}$$

where *ρ* is the density; *H* is the enthalpy; *t* is the time; and *k* is the thermal conductivity of the material; *q_s_* and *q_v_* are the surface and volume heat source terms, respectively.

With Equation (2), most thermal processes can be described mathematically. According to the specific problem, additional boundary conditions must be included as follows:

The initial condition of the model at *t* = 0 is a uniform temperature distribution, which is

(3) $$T\left(x,y,z,0\right)=T_{0}\left(x,y,z\right)=35\;{}^{\circ }C$$

where *T*_0_ is the ambient temperature.

The boundary condition on the top surface *z = z_top_* can be defined as

(4) $$-k{\left(\frac{\partial T}{\partial z}\right)}_{z=z_{top}}=q_{s}+q_{v}-h\left(T_{0}-T_{\mathrm{surf}}\right)$$

where *h* is the total heat transfer coefficient by radiation and convection into the ambient, and is reported to be 80 W/m^2^ K [24]. *T*_surf_ is the temperature of the powder bed surface.

The bottom plane and the other vertical walls are modeled to be thermally insulating.

### 2.3. Heat Source

**Figure 3:**
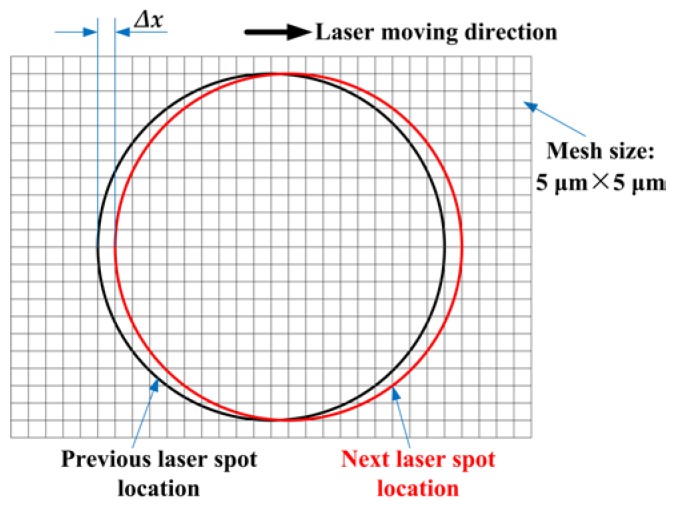
Representation of heat source movement.

The most widely adopted heat source in the laser metal PBF process is the Gaussian distribution model (TEM_00_), which is circularly symmetrical in cross section. The incident heat flux across the laser beam is defined as:

(5) $$q_{in}=\frac{2P}{\pi r_{b}^{2}}\exp\left[-2\frac{{\left(x-x_{s}\right)}^{2}+{\left(y-y_{s}\right)}^{2}}{r_{b}^{2}}\right]$$

where *P* is the laser power, *r_b_* is the radius of the laser beam at which the heat flux value is e^−2^ times of that of the laser beam center, and the point (*x_s_, y_s_*) is the position of the heat source. In some existing models [19,20], square spot with uniform distribution is used to reduce the model complexity. However, this cannot accurately represent the characteristics of Gaussian heat source. In this paper, a more accurately modeled laser spot is introduced, which is illustrated in Figure 2b. When the heat source moves from a step point (*x_s_^i^, y_s_^i^*) to the next step point (*x_s_^i^ + ∆x, y_s_^i^*) with a velocity (*V*) along the scanning direction (as shown in Figure 3), the radiative heat input over a time step into any cell centered around (*x*, *y*) is obtained through a space integration of Equation (5)

(6) $$Q_{cell}=t_{step}\times \int_{x-\Delta x/2}^{x+\Delta x/2}\int_{y-\Delta y/2}^{y+\Delta y/2}q_{in}dxdy$$

Then the total radiative heat input over a time step to the modeled spot can be calculated as the sum of input to all cells in Ω, as shown in Equation (7)

(7) $$Q_{in}=\sum_{\Omega }Q_{cell}$$

### 2.4. Absorption

During the laser metal PBF process, the material state and phase are changed from powder to liquid, and then to solid over the time horizon during which the laser beam irradiates the powder bed. At each material stage and phase, a different absorption mechanism is applied. At the beginning of the PBF process, the heat absorbed by metal powder is not sufficient to melt the top layer powder, which results in the laser beam penetrating through the powder due to the pores, and causes the energy of the laser beam to be attenuated through the powder bed. In other words, some of the input heat at this stage is absorbed by the powderbed and the substrate underneath the powder bed. But once the top layer powder is fully melted to dense liquid, the penetration mechanism of the laser beam into areas underneath the top layer (i.e., lower layer powder, substrate) is prevented by the metal liquid formed. In turn, the interaction between laser and metal liquid becomes the major issue influencing the overall light absorptance. Meanwhile, if multi-track with overlapping is considered, the laser beam will also interact with the previous-solidified track. Therefore, in the PBF process, the laser beam interacts with materials in various states, including the un-melted powder and its underlying substrate, the melted liquid, and the previous-solidified track, which is shown in Figure 1. In this figure, the surface area of each state covered by the laser spot is defined by using normalized parameters, *S_i_* (*i* = liquid or solid or powder or substrate). Obviously, the sum of *S_liquid_*, *S_solid_*, and *S_powder_* is equal to the laser spot area (Ω), and *S_substrate_* is the same as *S_powder_*.

For both the liquid and solid states, the heat source is assumed to be a surface heat source irradiating on surface elements, because the optical penetration depth of dense material is of the order of tens of nanometers [8,28]. Equation (5) gives the heat source, as a surface heat source *q_s_*, irradiating on a surface element of dense material with a fraction of heat absorbed (*A*) and reflected (*1-A*). The absorptance of the laser beam is different from solid (*A_solid_*) to liquid (*A_liquid_*) [33]. The absorptance of Ti in the solid state is nearly temperature independent and approximately constant [34]. Nevertheless, when melting takes place in an inert gas atmosphere, the material surface changes from an uneven powder surface to a flat and oxide-free liquid surface, which can lead to a sharp decrease of absorptance [35]. It is, however, extremely difficult to measure the absorptance of liquid [34], which is usually estimated at 10–20% for almost any metal exposed to a 1.06 μm laser beam [36]. In this work, *A_solid_* and *A_liquid_* for Ti6Al4V were set at 35% [37] and 12%, respectively. Therefore, the calculated heat absorbed by liquid within *S_liquid_* and by solid within *S_solid_* over a time step can be expressed in Equations (8) and (9), respectively

(8) $$Q_{liquid}=A_{liquid}\sum_{S_{liquid}}Q_{cell}$$

(9) $$Q_{solid}=A_{solid}\sum_{S_{solid}\;}Q_{cell}$$

In the case of material in a powder state, a laser beam penetrates into powder through pores to a depth of several particle diameters due to a multiple reflections effect [28,38]. The radiation transfer equation in the direction of laser thickness presented by Gusarov et al. [39] is used in this work. This radiation transfer treats the powder bed with a defined thickness *z_bed_* as an optical medium with an extinction coefficient *β*. The laser energy flux through the powder bed *q* can be expressed by

(10) $$\frac{q}{q_{in}}=\frac{Ra}{\left(4R-3\right)D}\{\left(1-R^{2}\right)e^{-\lambda }\left[\left(1-a\right)e^{-2a\xi }+\left(1+a\right)e^{2a\xi }\right]-\left(3+Re^{-2\lambda }\right)\times \;\left\{\left[1+a-R\left(1-a\right)\right]e^{2a\left(\lambda -\xi \right)}+\left[1-a-R\left(1+a\right)\right]e^{2a\left(\xi -\lambda \right)}\right\}\}-\frac{3\left(1-R\right)\left(e^{-\xi }-Re^{\xi -2\lambda }\right)}{4R-3}$$

where *λ = βz_bed_* is the optical thickness of the powder bed, *ξ = βz* is the dimensionless local depth coordinate, $a=\sqrt{1-R}$ and *D* is given by

(11) $$D=\left(1-a\right)\left[1-a-R\left(1+a\right)\right]e^{-2a\lambda }-\left(1+a\right)\left[1+a-R\left(1-a\right)\right]e^{2a\lambda }$$

Assuming that the powder bed consists of spherical particles of diameter *d_p_*, *β* can be given by

(12) $$\beta =\frac{3}{2}\frac{1-\phi }{\phi }\frac{1}{d_{p}}$$

where *φ* is the powder bed porosity, which is generally in the range 40–60% for typical metallic powders [39]. The fraction of the laser energy flux absorbed by the substrate is then given by the *z = z_bed_* of Equation (10)

(13) $$\frac{q}{q_{in}}\left(\;z=z_{bed}\right)=\frac{Ra}{\left(4R-3\right)D}\{\left(1-R^{2}\right)e^{-\lambda }\left[\left(1-a\right)e^{-2a\lambda }+\left(1+a\right)e^{2a\lambda }\right]-\;2\left(1-R\right)\left(3+Re^{-2\lambda }\right)\}-\frac{3{\left(1-R\right)}^{2}e^{-\lambda }}{4R-3}$$

**Figure 4:**
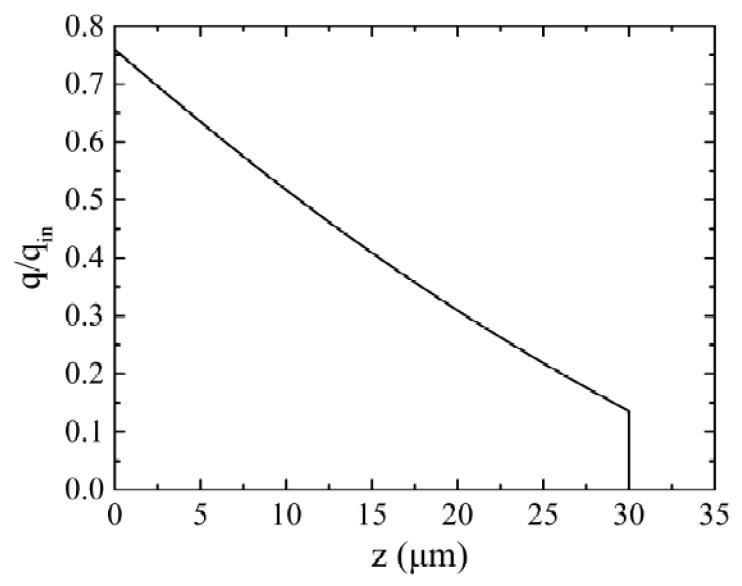
The ratio of laser energy flux through the powder bed to incident heat flux versus the distance *z* from the top surface for a layer of 30 μm on a solid substrate.

Compared with the dense material, the material in a powder state has a higher absorptance [28,38]. The absorptance of powder is nearly temperature independent as well [40]. The possible reason is that when no melting occurs, there is no change in the surface structures of the powder bed. For the case considered in this work, namely a powder layer of 30 μm of Ti6Al4V powder with a porosity of 50% and a mean diameter of 30 μm on a substrate of dense Ti6Al4V, the ratio of laser energy flux through the powder bed to the incident heat flux *q/q_in_* is shown in Figure 4. From this figure, it is implied that about 76% of incident laser energy is absorbed by the powder and underlying substrate, in which approximately 62% is absorbed by the powder, and about 14% by the substrate.

The heat source as a volumetric heat source penetrating into the powder bed due to multiple reflections effect can be obtained by

(14) $$q_{v}\left(x,y,z\right)=-\frac{dq}{dz}$$

The calculated heat input over a time step into any one powder element centered around (*x*, *y*, *z*) is given by a space integral of Equation (14)

(15) $$Q_{powder}^{elem}=t_{step}\times \int_{x-\Delta x/2}^{x+\Delta x/2}\int_{y-\Delta y/2}^{y+\Delta y/2}\int_{z-\Delta z/2}^{z+\Delta z/2}q_{v}\left(x,y,z\right)dxdydz$$

Then the heat absorbed by powder elements within the volume range of *S_powder_ × z_bed_* over a time step can be obtained by

(16) $$Q_{powder}=\sum_{S_{powder}\times z_{bed}}Q_{powder}^{elem}$$

The calculated heat input over a time step on the surface of any one substrate element centered around (*x, y, z_bed_*) is obtained through a space integration of Equation (13)

(17) $$Q_{substrate}^{elem}=t_{step}\times \int_{x-\Delta x/2}^{x+\Delta x/2}\int_{y-\Delta y/2}^{y+\Delta y/2}q\left(x,y,z_{bed}\right)dxdy$$

Then the heat absorbed by substrate elements within *S_substrate_* over a time step can be given by

(18) $$Q_{substrate}=\sum_{S_{substrate}}Q_{substrate}^{elem}$$

The heat absorbed by the elements in all cases considered over a time step can be calculated by

(19) $$Q_{abs}=Q_{liquid}+Q_{solid}+Q_{powder}+Q_{substrate}$$

If both sides of Equation (19) are divided by *Q_in_*, a new relationship can be expressed as

(20) $$\frac{Q_{abs}}{Q_{in}}=\frac{Q_{liquid}}{Q_{in}}+\frac{Q_{solid}}{Q_{in}}+\frac{Q_{powder}}{Q_{in}}+\frac{Q_{substrate}}{Q_{in}}$$

Obviously, the left part of Equation (20) (*Q_abs_/Q_in_*) is the definition of the absorptance, which is the ratio of the amount of absorbed heat to the input heat at laser spot location in a time step. Because the time step is very small, this absorptance can be regarded as instantaneous absorptance, here expressed by a symbol of *A_instant_* (i.e., *A_instant_* = *Q_abs_/Q_in_*). For the right side of Equation (20), it can be interpreted as contribution of each state and phase to *A_instant_*. Normalized parameters, *C_i_*, are used to represent each contribution.

(21) $$C_{i}=\frac{Q_{i}}{Q_{in}},$$

i=liquid or solid or powder or substrate. It can be deduced from the above formulae that *C_i_* is determined by *S_i_*, which in turn affects *A_instant_*.

### 2.5. Material Properties

A detailed definition of material properties, which are related to the temperature and the transition of the material from powder to liquid and then to solid, is the basis for performing accurate simulations of the laser metal PBF process. The thermal properties of most of the standard materials, such as density, thermal conductivity, specific heat, enthalpy, and so on, are typically available within literature. However, most of the values found in literature correspond to the dense material, few values for powder are found. Nevertheless, several models have been proposed for the relationship between the powder and dense material [11,22].

The powder density *ρ_powder_* and effective thermal conductivity *k_eff_* can be written as [11]

(22) $$\rho_{powder}=\{\begin{array}{l} \left(1-\phi \right)\rho_{dense}\left(T\right),\;\;\;\;T_{0}\leq T\leq T_{s}\\ \left(1+\phi \frac{\left(T-T_{s}\right)}{\left(T_{l}-T_{s}\right)}-\phi \right)\rho_{dense}\left(T\right),\;\;\;\;\;T_{s}<T<T_{l\;\;\;}\\ \rho_{dense}\left(T\right),\;\;\;\;T\geq T_{l} \end{array}$$

(23) $$k_{eff}=\{\begin{array}{l} k_{powder}\left(T\right),T_{0}\leq T\leq T_{s}\\ \frac{k_{dense}\left(T_{l}\right)-k_{dense}\left(T_{s}\right)}{\left(T_{l}-T_{s}\right)}\left(T-T_{s}\right)+k_{powder}\left(T_{s}\right),\;\;\;\;\;T_{s}<T<T_{l}\\ k_{dense}\left(T\right),\;\;T\geq T_{l} \end{array}$$

where *φ* = 50% is the powder porosity; *T_0_* = 35 °C is the initial temperature of the powder; *ρ_dense_*(*T*) and *k_powder_*(*T*) are the density of the dense material and the thermal conductivity of the powder at the temperature *T*, respectively; *k_dense_*(*T_s_*) and *k_dense_*(*T_l_*) are the thermal conductivity of the dense material at the solidus temperature *T_s_* and the liquidus temperature *T_l_*, respectively.

The thermal conductivity of powder is mainly determined by inert gas in the pores and not so much by the properties of the material. It has been reported that the thermal conductivity is typically from 0.1 to 0.3 (W/m K) from room temperature to solidus temperature [19]. The thermal conductivity of powder is therefore modeled linearly, increasing from 0.1 to 0.3 (W/m K).

The material used in this model is titanium alloy Ti6Al4V. Based on these formulae and the thermo-physical properties of material in the dense state [41,42] summarized in Table 1, *ρ_powder_* and *k_eff_* for the Ti6Al4V powder can be obtained.

### 2.6. Numerical Setup

In order to investigate the effects of the time of laser radiation, as well as process parameters on instantaneous absorptance, numerical calculations have been performed using the ANSYS Multiphysics FE package at different parameter sets, which are listed in Table 2. The length and height of the 3D FE model are 640 μm and 180 μm, respectively. Here, the width depends on the chosen hatch space. In this model, eight-noded hexahedron elements with a fine-and-coarser mesh style are used, namely the scanned region with fine mesh and the surrounding loose powder and substrate with coarser mesh. The fine mesh size is chosen so that reduction does not alter the results significantly and is taken as 5 μm. Thus, for a given mesh size, the time step sizes at scanning velocity V = 0.25 m/s and V = 1.25 m/s (in Table 2) are 2 × 10^−5^ s and 4 × 10^−6^ s, respectively. The track length with 400 μm is sufficient to obtain a balanced material state at laser spot location. The element and node numbers at three parameter sets (in Table 2) are different, which are controlled by the size of the chosen hatch space and the number of tracks. For the process parameter set I, the element and node numbers are 110,144 and 119,544 respectively, and its computation time is about 12 h running on 8 cores.

## 3. Results and Discussion

### 3.1. Simulation Validation

**Figure 5:**
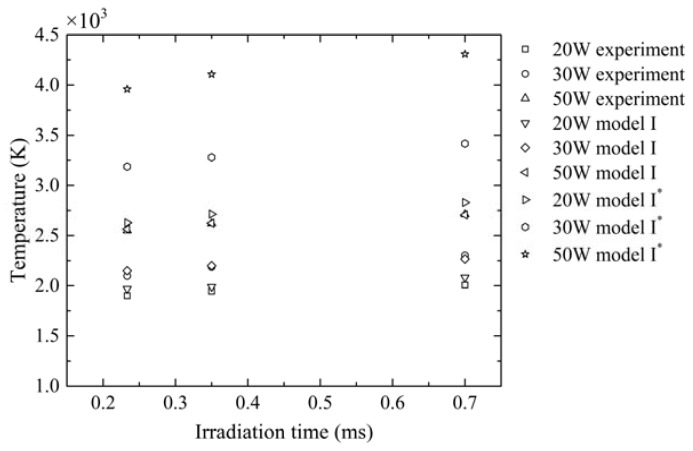
Comparison of the peak temperature in the simulation and in the experiment. Irradiation time is the ratio of laser spot diameter to scanning velocity.

**Figure 6:**
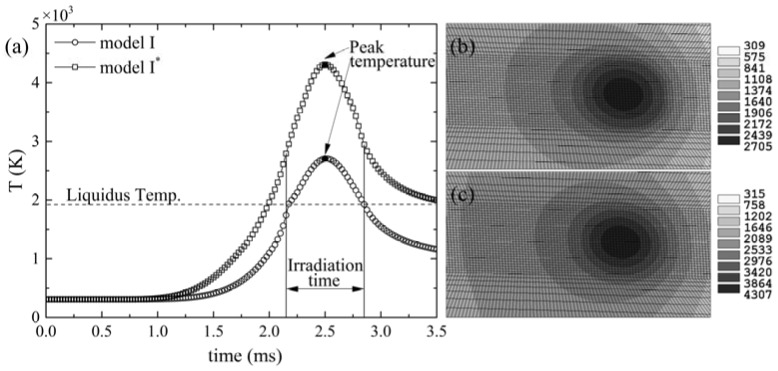
(**a**) Temperature history for a given point which is a distance of 250 μm away from the start point; (**b**,**c**) temperature distribution (unit in K) from the model with and without considering the effect of material state and phase changes on laser absorption, respectively. *P* = 50 W, *V* = 0.1 m/s, *r_b_* = 35 μm.

It is known that laser absorption of material affects the temperature distribution which influences the thermal stress development and the final quality of parts. In order to determine the suitability of the model in the temperature simulation of a laser metal PBF process, comparative studies with experimental work were conducted. Figure 5 compares the simulated peak temperatures in both options, with and without considering the effect of material state and phase changes on laser absorption, with the published experimental measurements by Yadroitsev et al. [43] in the peak temperature at the substrate Ti6Al4V without powder. The process parameters of the experiment are listed in Table 3. Figure 5 shows that the maximum temperatures predicted by the model considering the effect of material state and phase changes on laser absorption (i.e., model I) showed a better agreement with the experimental data than that by the existing model without considering (i.e., model I*). In the former scenario, phase change was accompanied by absorptance change, leading to a decrease of the total absorptance of the material, and in turn causing the heating rate to decrease after the temperature exceeded the liquidus line, and the transient temperature of material was always lower than that of model I*, as shown in Figure 6. While in the case of model I*, ignoring of the effect of the material state and phase changes on laser absorptance led to a high heating rate and a relatively high peak temperature. Therefore, the change in laser absorption as a result of the state and phase change had a significant effect on the temperature.

### 3.2. Instantaneous Absorptance Evolution

#### 3.2.1. Instantaneous Absorptance Evolution with Time and Track

**Figure 7:**
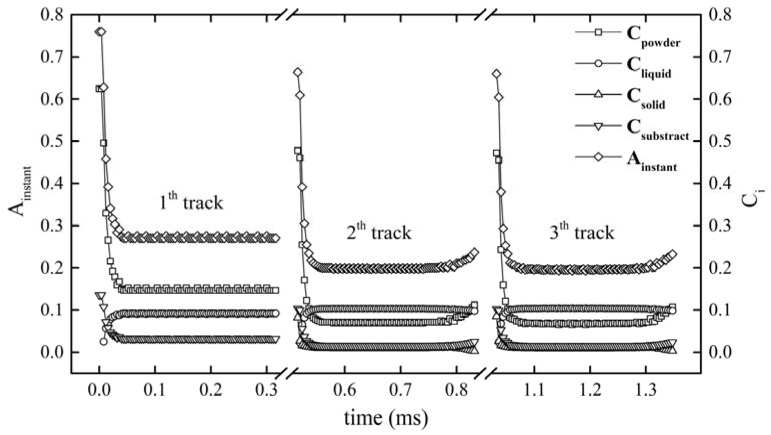
The evolution of *A_instant_* and *C_i_* with the development of time and track at the parameter set I.

**Figure 8:**
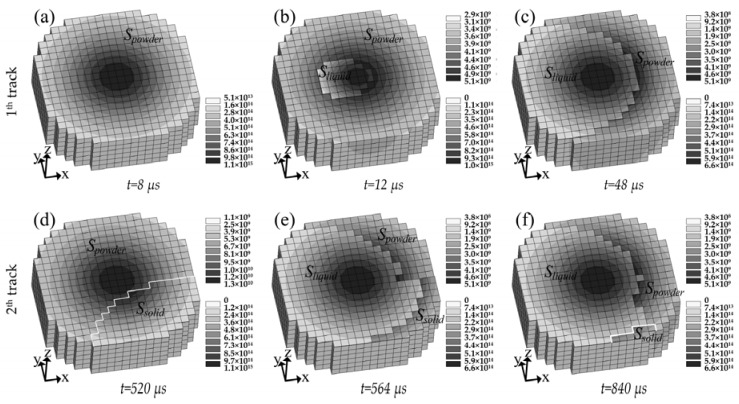
Area distribution of the powder, solid, and liquid state material and their absorbed energy density distribution at laser spot location at various times and tracks of Figure 7. The top right legend corresponds to the material in the dense state (unit in J m^−2^s^−1^), while the bottom right legend corresponds to the material in the powder state (unit in J m^−3^s^−1^).

Figure 7 shows the change of *A_instant_* and *C_i_* with the laser beam moving from the start point of the first track to the end point of the third. Figure 8 shows the area distribution of the powder, solid, and liquid state material and their absorbed energy density distribution under the area of laser spot (Ω) at various times and tracks of Figure 7. It can be seen that *A_instant_* maintains a high value at the beginning of the laser irradiating the first track. This is because the laser-irradiated area of the powder had not melted and still remained in powder state, as shown in Figure 8a, which has a high absorption capacity and hence results in high *A_instant_*. With the laser beam further irradiating the powder bed, *S_powder_* decreased while a small area of dense liquid occurred near the center of the laser spot, which is shown in Figure 8b. Subsequently *S_liquid_* broadened quickly in the direction opposite to the scanning direction, while slightly in the scanning direction, which was due to the moving laser causing fresh powder to be scanned. Finally, both *S_liquid_* and *S_powder_* reached a stable state, as shown in Figure 8c. Correspondingly, the decrease of *A_instant_* is interpreted as a consequence of the decrease of *S_powder_*. When the first track was finished, the laser spent 200 μs to switch off and jump to the start point of the second track. As such the first track was cooled down to be solid. Thanks to this reason and the selection of a smaller hatch space, at the beginning of irradiating the second track, the laser exposure area covered part of solid (*S_solid_*), which is shown in Figure 8d. In turn, because *S_powder_* at the beginning of the second track was smaller than that of the first track, the starting point of *A_instant_* was smaller than that of the first track as shown in Figure 7. Different from the first track, the *A_instant_* at the end of the second track had a slight rebound. Such a rise was caused by the “Semicircle” shape at the end of the previous solidified track, leading to the increase of *S_powder_* with the moving of the laser beam to the end of the second track, which is shown in Figure 8f. Since the evolution of *A_instant_* in the third track was similar to that in the second track, it is not elaborated here.

From the above analysis, it is indicated that *A_instant_* stays at a high value at the beginning of each track and decreases dramatically with the laser irradiation, which is due to the change of *C_i_* as a result from *S_i_*. Such a decline was also observed in the experimental study [28] based on the absorptance measurements on the Maraging steel and the pure silver in SLM, respectively, which shows that *A_instant_* decreases with the laser exposure time and then reaches a stable value. Note that a high *A_instant_* at the beginning of each track can cause over-heating and an initial bump.

#### 3.2.2. Instantaneous Absorptance Evolution with Process Parameters

**Figure 9:**
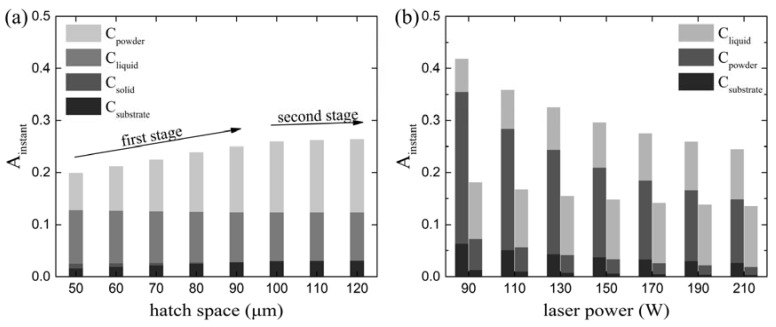
(**a**) *A_instant_* in the stable state versus hatch space at the parameter set II. (**b**) The *A_instant_* in the stable state versus laser power at the parameter set III, where the left column is at scanning velocity 1.25 m/s, and the right is at 0.25 m/s.

**Figure 10:**
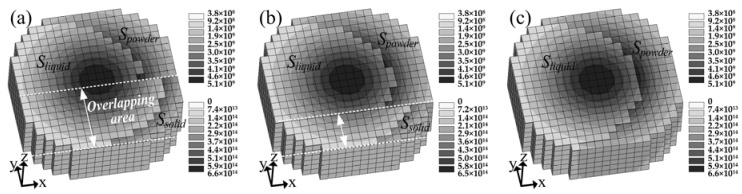
Area distribution of each state and their absorbed energy density distribution under the laser spot area with hatch space of (**a**) 50 μm; (**b**) 70 μm; and (**c**) 100 μm.

It is shown in Figure 7 that *A_instant_* starts from a high value and then decreases dramatically, and finally keeps at a relatively stable value for a long time period in a track. Thus, the stable-value period of *A_instant_* within a track must be emphasized in order to investigate the effects of different process parameters on *A_instant_*. Figure 9 shows the change of *A_instant_* in a stable-value period with the change of hatch space, scanning velocity, and laser power, respectively. Figure 9a contains two stages of hatch space, including hatch space less than 100 μm and over 100 μm. Throughout the first stage (*HS* < 100 µm), *A_instant_* had a gradual increase from 0.199 to 0.260 alongside the increase of hatch distance from 50 μm to 100 μm. A similar result was observed in experimental investigations [29,30,31]. In the second stage (*HS* ≥ 100 µm), although the hatch space increases from 100 μm to 120 μm, *A_instant_* keeps basically constant (a slight increase from 0.260 to 0.264). In order to assure the quality properties of the connection areas between two consecutive tracks in PBF, overlapping area between two consecutive tracks was applied in the PBF process, which resulted in overlapping area being re-melted, as depicted in Figure 10a. Actually, this overlapping area was a part of the previous solidified track. When the laser beam scanned the second track, the overlapping area was initially solid, then a part of the overlapping area changed from solid to liquid, and the remaining area still kept as solid. Eventually, the liquid area and *S_solid_* in the overlapping area reached a stable state, which is also shown in Figure 10a. Meanwhile, it is worthy to note that the overlapping area decreased with the increase of hatch space from 50 μm (Figure 10a) to 70 μm (Figure 10b), and then vanished when the hatch space reached 100 μm (Figure 10c), which resulted in that both *S_solid_* and *S_liquid_* decreased, but *S_powder_* increased, and then *S_solid_* vanished, and both *S_liquid_* and *S_powder_* reached a stable state. As such, both *C_solid_* and *C_liquid_* decreased, but *C_powder_* increased, and then *C_solid_* become zero and both *C_liquid_* and *C_powder_* maintained a stable value. However, the increment of *C_powder_* was larger than the sum decrement of *C_solid_* and *C_liquid_*, which was due to the fact that powder has a higher absorption capacity than liquid and solid. As a result, *A_instant_* increased with the increase of hatch space in the first stage, and remained basically constant in the second stage. The slight increase of *A_instant_* in the second stage may be caused by the effect of the previous-scanned preheating. The larger the hatch space is, the smaller the effect of preheating will be, which makes the powder take more time to turn into liquid.

**Figure 11:**
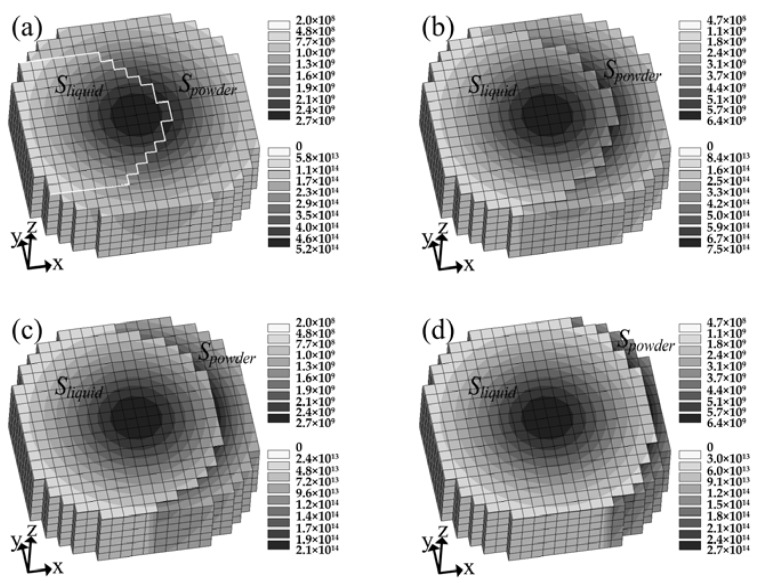
Area distribution of each state and their absorbed energy density distribution under the laser spot at different laser power and scanning velocity: (**a**) *P* = 90 W, *V* = 1.25 m/s; (**b**) *P* = 210 W, *V* = 1.25 m/s; (**c**) *P* = 90 W, *V* = 0.25 m/s; (**d**) *P* = 210 W, *V* = 0.25 m/s.

It can be seen from Figure 9b that *A_instant_* increased with the growth of scanning velocity, but decreased with the growth of laser power. These results are consistent with trends found in the previous experimental studies [29,30,31]. For a fixed laser power, as the scanning velocity increases, the heat absorbed by the powder in a time step decreases, making it harder to be melted. Thus, *S_powder_* increases but *S_liquid_* decreases when the scanning velocity changes from 0.25 m/s (Figure 11c,d) to 1.25 m/s (Figure 11a,b), which results in *C_powder_* increasing but *C_liquid_* decreasing, which is shown in Figure 9b. Therefore, *A_instant_* increases with the increase of the scanning velocity. It is worth noting that *A_instant_* will maintain a high constant value as the laser moves at a sufficient high velocity [30], owing to that the absorbed heat is not enough to melt the powder. On the other hand, for a fixed scanning velocity, the heat absorbed by the powder in a time step increases with the increase of the laser power, making it easier to be melted. As such, *S_powder_* decreases but *S_liquid_* increases with the increase of laser power from 90 W (Figure 11a,c) to 210 W (Figure 11b,d), leading to *C_powder_* decreasing but *C_liquid_* increasing, as shown in Figure 9b. Therefore, *A_instant_* decreases with increasing the laser power. In addition, it can be seen from Figure 9b, *A_instant_* declines more quickly at a high scanning velocity than a low scanning velocity. It is known that the energy density of laser beam with Gaussian distribution decreases from laser spot center to radius area. Thus, for a higher scanning velocity, the decrement of *S_powder_* or increment of *S_liquid_* caused by the increase of laser power is irradiated by relatively higher energy density in the Gaussian distribution, which gives rise to a bigger decline ratio of *A_instant_*.

## 4. Conclusions

A three-dimensional finite element model of heat transfer considering the effect of material state and phase changes on laser absorption is presented to gain insight into the absorption mechanism and evolution of instantaneous absorptance during the PBF process. The model is also used in the simulation of temperature field to determine its suitability, and a comparative study of the simulated peak temperatures with experimental data is conducted. The following conclusions are drawn.

(1) Compared to the existing model without considering the effect of material state and phase changes on laser absorption, the simulated peak temperatures from the model with considering the effect of material state and phase changes on laser absorption are in high agreement with experimental data. In addition, absorption change due to material state and phase change has a great effect on temperature.
(2) The instantaneous absorptance evolves with the time of laser irradiation and track. The instantaneous absorptance initially stays a high value and then decreases to a stable value with the time of laser irradiation for each track, and rebounds a little at the end of each track except from the first track.
(3) The instantaneous absorptance in a stable-value period elevates from 0.199 to 0.260 as the hatch space increases from 50 μm to 100 μm, while keeping basically constant as the hatch space further increases from 100 μm to 120 μm.
(4) The instantaneous absorptance of the first track in a stable-value period increases with increasing the scanning velocity, but decreases with increasing the laser power. Moreover, the instantaneous absorptance drops more significantly with increasing the laser power at a high scanning velocity than at a low scanning velocity.

## Figures and Tables

**Table 1:** Summary of material thermo-physical properties of Ti6Al4V [41,42].

*T* (K)	298	573	873	1253	1283	1473	1877	1923
*k* (W/m K)	7	10.15	14.2	22.7	19.3	22.9	27	33.4
*ρ* (kg/m^3^)	4420	4381	4336	4282	4282	4252	4198	3920
*H* (J/g)	0	158	350	636	684	816	1102	1466
*T_s_* (K)	1877							
*T_l_* (K)	1923							

**Table 2:** FEM process parameters.

Set No.	Laser Power *P* (W)	Scanning Velocity *V* (m/s)	Number of Tracks	Hatch Space *HS* (μm)	Layer Thickness *z_bed_* (μm)	Spot Radius *r_b_* (μm)
Set I	170	1.25	3	50	30	50
Set II	170	1.25	2	50–120 (Increment: 10)		
Set III	90–210 (Increment: 20)	0.25	1	-		
		1.25				

**Table 3:** Parameters in the experiment of Yadroitsev et al. [43].

Parameter	Notation	Value
Laser power (W)	*P*	20, 30, 50
Scanning velocity (m/s)	*V*	0.1, 0.2, 0.3
Spot radius (μm)	*r_b_*	35
